# Apolipoprotein B and interleukin 1 receptor antagonist: reversing the risk of coronary heart disease

**DOI:** 10.3389/fendo.2023.1278273

**Published:** 2023-10-24

**Authors:** Fangkun Yang, Ning Huangfu, Jiaxi Shen, Pengpeng Su, Lujie Zhu, Hanbin Cui, Shuai Yuan

**Affiliations:** ^1^ Department of Cardiology, First Affiliated Hospital of Ningbo University (Ningbo First Hospital), School of Medicine, Ningbo University, Ningbo, China; ^2^ Key Laboratory of Precision Medicine for Atherosclerotic Diseases of Zhejiang Province, Ningbo, China; ^3^ Cardiovascular Disease Clinical Medical Research Center of Ningbo, Zhejiang, China; ^4^ School of Medicine, Wenzhou Medical University, Wenzhou, China; ^5^ Unit of Cardiovascular and Nutritional Epidemiology, Institute of Environmental Medicine, Karolinska Institutet, Stockholm, Sweden

**Keywords:** apolipoprotein B, interleukin 1 receptor antagonist, coronary heart disease, myocardial infarction, lifestyle modification

## Abstract

**Aims:**

Epidemiological evidence for the link of interleukin 1 (IL-1) and its inhibition with cardiovascular diseases (CVDs) remains controversial. We aim to investigate the cardiovascular effects of IL-1 receptor antagonist (IL-1Ra) and underlying mechanisms.

**Methods:**

Genetic variants identified from a genome-wide association study involving 30,931 individuals were used as instrumental variables for the serum IL-1Ra concentrations. Genetic associations with CVDs and cardiometabolic risk factors were obtained from international genetic consortia. Inverse‐variance weighted method was utilized to derive effect estimates, while supplementary analyses employing various statistical approaches.

**Results:**

Genetically determined IL-1Ra level was associated with increased risk of coronary heart disease (CHD; OR, 1.07; 95% CI: 1.03-1.17) and myocardial infarction (OR, 1.13; 95% CI: 1.04-1.21). The main results remained consistent in supplementary analyses. Besides, IL-1Ra was associated with circulating levels of various lipoprotein lipids, apolipoproteins and fasting glucose. Interestingly, observed association pattern with CHD was reversed when adjusting for apolipoprotein B (OR, 0.84; 95%CI: 0.71-0.99) and slightly attenuated on accounting for other cardiometabolic risk factors. Appropriate lifestyle intervention was found to lower IL-1Ra concentration and mitigate the heightened CHD risk it posed.

**Conclusion:**

Apolipoprotein B represents the key driver, and a potential target for reversal of the causal link between serum IL-1Ra and increased risk of CHD/MI. The combined therapy involving IL-1 inhibition and lipid-modifying treatment aimed at apolipoprotein B merit further exploration.

## Introduction

Coronary heart disease (CHD) represents the leading cause of morbidity and mortality worldwide, posing a significant public health concern (1). Recent research has characterized CHD as a chronic inflammatory disease with inflammation-mediated atherosclerosis being a key contributing factor, which sparks interests in the potential benefits of targeting inflammation in cardiovascular diseases (CVDs) (2). Interleukin 1 (IL-1), a prototypical proinflammatory cytokine, plays a pivotal role in the innate immune response by activating the IL-1 receptor and thus leading to the activation of downstream inflammatory mediators. IL-1 pathway has emerged as a promising therapeutic target for rheumatoid arthritis (RA) and CVDs (3). Nevertheless, the question remains whether targeting IL-1 related inflammation could translate into clinical benefit against cardiovascular events.

IL-1 receptor antagonist (IL-1Ra) provides natural inhibition by competing with IL-1α and IL-1β for binding to the receptor (4). Despite its inhibitory role, the epidemiological evidence remains controversial. A meta-analysis comprising six population-based cohorts has revealed that levels of serum IL-1Ra exhibit a positive correlation with the risk of developing CVDs (5). However, a prospective cohort study of over 800 CHD-free individuals found no association between circulating IL-1Ra and the risk of CHD (6). The causality of this association remains shrouded in uncertainty, primarily due to the influence of confounding factors and the potential for reverse causation.

Anakinra is a human recombinant IL1-Ra and a first-line agent for RA, however, its cardiovascular effects remain unclear (7). In a randomized controlled trial (RCT) involving 80 patients with RA, Anakinra was found to significantly improve various vascular function indices as well as myocardial deformation and twisting (8). However, another RCT of 182 patients with non-ST elevation acute coronary syndrome suggested that treatment with Anakinra increased the risk of major adverse cardiovascular events at one year of follow-up (9). Notwithstanding, the duration and statistical power of previous trails have fallen short in evaluating the effect on cardiovascular outcomes.

An alternative strategy focusing on the genetic variants responsible for the inhibition of IL-1 could strengthen the causal inference (10). Utilizing genetic variants randomly assigned at conception as an instrumental variable (IV), it is possible to derive estimates that are less susceptible to environmental confounding factors, measurement error, and reverse causality (11). Our previous study has indicated the potential effect of lifetime exposure to elevated levels of IL-1Ra on an increased CHD risk, but the underlying mechanisms were not fully elucidated (12). Another early Mendelian randomization (MR) study suggested that long-term dual IL-1α/β inhibition increased concentrations of LDL-cholesterol, but not apolipoprotein or glycaemic traits (13). In recent years, the genetic determinants of IL-1Ra and cardiometabolic risk factors have been further well-characterized, which provides a promising tool to assess the causal network. In addition to pharmacological interventions, lifestyle modification represents a crucial aspect of clinical management. An investigation into the interplay between various lifestyle factors and the levels of IL-1Ra could provide insights into the heightened risk of developing CVDs.

Thus, we utilized a genetic approach to mimic the causal effects of serum IL-1Ra on the risks of RA and five major CVDs, including CHD, myocardial infarction (MI), heart failure (HF), atrial fibrillation (AF) and ischemic stroke (IS), as well as circulating lipoprotein lipids, apolipoproteins, glycaemic traits, and blood pressure to gain insights into underlying mechanisms. Furthermore, the causal associations of obesity and lifestyle factors with IL-1Ra concentrations were examined.

## Methods

### Study design

**Figure 1:**
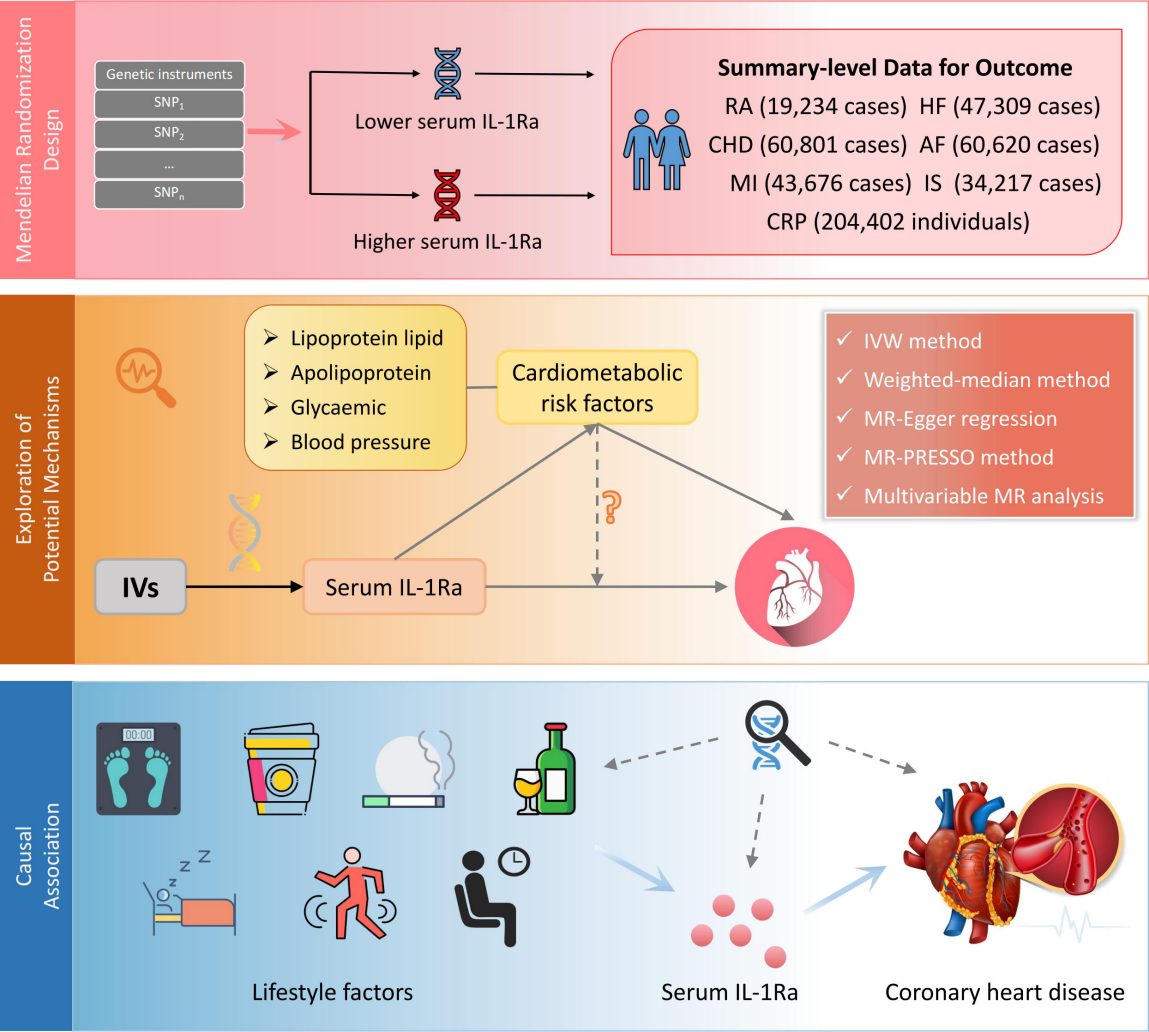
Design of the current two-sample Mendelian randomization study. IV, instrumental variable; IL1-Ra, interleukin 1 receptor antagonist; CHD, coronary heart disease; MI, myocardial infarction; HF, heart failure; AF, Atrial fibrillation; IS, ischemic stroke; RA, rheumatoid arthritis; CRP, C-reactive protein.

A comprehensive study was designed to investigate the causal role of IL-1Ra (**Figure 1**). First, we selected genetic variants as an IV for serum concentrations of IL-1Ra. Second, the combined effects of such genetic variants on RA and circulating concentrations of C-reactive protein (CRP) were assessed to examine whether the genetic IVs were valid and robustly linked with IL-1Ra concentrations. Then, the causal associations of genetically predicted IL-1Ra concentrations with the risk of five major CVDs (CHD, MI, HF, AF and IS) were investigated. Third, the exploratory analysis of IL-1Ra concentrations in relation to main cardiometabolic risk factors were performed to better understand potential mechanisms between long-term pharmacological IL-1 inhibition and CHD/MI risk. Fourth, based on the above analysis, the supplementary analyses were further conducted with the adjustment of risk factor that was closely linked with IL-1Ra concentrations. Fifth, we assessed the effects of common lifestyle factors on the serum concentrations of IL-1Ra and compared the causal effect sizes of lifestyle factors on the risk of CHD with or without the adjustment of IL-1Ra concentrations.

### Genetic instrument selection

Genetic variants associated with serum concentrations of IL-1Ra were identified from a genome-wide association study (GWAS) involving 30,931 individuals of European descent across 15 studies (**Table 1**) (14). The primary study utilized the Olink proximity extension assay cardiovascular I panel to measure 90 cardiovascular-related proteins (14). Single nucleotide polymorphisms (SNPs) for serum concentrations of IL-1Ra were identified from genetic variants in the *IL1RN* gene ± 5 Mb region at the genome-wide significance threshold (*p* < 5×10^−8^), followed by linkage disequilibrium pruning based on the European 1000 Genomes Project reference panel (*r* ^2^ < 0. 1; clump distance > 10000 kb) (15). Among SNPs in linkage disequilibrium, the one with the smallest *p* was retained. Thirteen independent SNPs were selected as a genetic IV with an effect size scaled to one standard-deviations (SD) increase in IL-1Ra concentrations (**Supplementary Table 1**).

**Table 1:** Detailed information on data sources.

Trait	Participants	Ancestry	Adjustments	Unit	PubMed ID
IL-1Ra	30,931 individuals	European	age, sex and 1-10 principal components	SD	33067605
Rheumatoid arthritis	19,234 cases and 61,565 controls	European	top 5-10 principal components	Odds ratio	24390342
C-reactive protein	204,402 individuals	European	age, sex, and population substructure	SD	30388399
Coronary heart disease	60,801 cases and 123,504 controls	Mixed	Not reported	Odds ratio	26343387
Myocardial infarction	43,676 cases and 128,199 controls	Mixed	Not reported	Odds ratio	26343387
Heart failure	47,309 cases and 930,014 controls	European	age, sex, the first 10 principal components	Odds ratio	31919418
Atrial fibrillation	60,620 cases and 970,216 controls	European	sex, age, age (2), and 4 principal components	Odds ratio	30061737
Ischemic stroke	34,217 cases and 404,630 controls	European	age and sex	Odds ratio	29531354
Apolipoprotein A-I	393,193 individuals	European	age, sex, BMI, genotyping chips	SD	32203549
Apolipoprotein B	439,214 individuals	European	age, sex, BMI, genotyping chips	SD	32203549
HDL	403,943 individuals	European	age, sex, BMI, genotyping chips	SD	32203549
LDL	440,546 individuals	European	age, sex, BMI, genotyping chips	SD	32203549
TG	441,016 individuals	European	age, sex, BMI, genotyping chips	SD	32203549
FG	281,416 individuals	European	age, sex and the first 5 principal components	SD	34059833
FI	213,650 individuals	European	age, sex and the first 5 principal components	SD	34059833
HbA1C	215,977 individuals	European	age, sex and the first 5 principal components	SD	34059833
SBP	up to 1,006,863 individuals	European	age, sex, BMI, genotyping chips	SD	30224653
DBP	up to 1,006,863 individuals	European	age, sex, BMI, genotyping chips	SD	30224653

IL1-Ra, interleukin 1 receptor antagonist; HDL, high density lipoprotein; LDL, low density lipoprotein; TG, total triglyceride; FG, fasting glucose; FI, fasting insulin; SBP, systolic blood pressure; DBP, diastolic blood pressure; SD, standard deviation.

### Data sources

Summary estimates of genetic associations with CHD and MI were extracted from the CARDIoGRAMplusC4D (Coronary ARtery DIsease Genome wide Replication and Meta-analysis (CARDIoGRAM) plus The Coronary Artery Disease (C4D) Genetics) consortium (**Table 1**) (16). Specifically, the study included ~185,000 participants, comprising 60,801 CHD cases and 123,504 controls, which were from 48 different cohort studies. The CHD cases were defined as acute coronary syndrome, chronic stable angina, coronary stenosis (over 50%) or MI (16). The majority (~80%) were of European ancestry and more than 70% of the total CHD cases had a history of MI (16). Similarly, the genetic associations with RA, CRP, HF, AF and IS were obtained from the corresponding GWASs (17–21). Detailed information was provided in the **Table 1**.

To explore the potential mechanisms, we obtained genetic associations with 10 main cardiometabolic risk factors, including apolipoprotein A-I, apolipoprotein B, high-density lipoprotein cholesterol (HDL-C), low-density lipoprotein cholesterol (LDL-C), and triglyceride (TG); fasting glucose (FG), fasting insulin (FI) and haemoglobin A1c (HbA1c); as well as systolic blood pressure (SBP) and diastolic blood pressure (DBP) (**Table 1**) (22–24).

Given the significance of lifestyle intervention in clinical management, genetic associations with various lifestyle factors, including obesity (measured by body mass index and waist circumference), smoking initiation, lifetime smoking index, alcohol drinking, alcohol dependence, coffee consumption, caffeine consumption, physical activity (both moderate-to-vigorous and vigorous), sedentary behaviour, sleep duration and insomnia were obtained from corresponding international consortia (**Supplementary Table 2**) (25–35).

All studies included in the GWAS had obtained ethical approval from the appropriate committees and written informed consent had been obtained from all participants. The data used in the current study were publicly available.

### Statistical analysis

The multiplicative random-effects inverse-variance-weighted (IVW) method, as the primary statistical model, was employed to estimate the associations of IL-1Ra concentrations with the risk of RA and CVDs, as well as the circulating concentrations of CRP (36). In addition, some supplementary analyses were conducted to investigate potential horizontal pleiotropy and assess the consistency of primary results, including the maximum likelihood method, weighted-median method, MR-Egger regression, and MR Pleiotropy Residual Sum and Outlier (MR-PRESSO) methods (37–39). The weighted median method represents a dependable approach for obtaining consistent causal estimates when up to 50% of the weight arises from the invalid instruments (37). The MR-Egger regression can detect the potential pleiotropy leveraging the embedded intercept test while also provide a corrected estimate accounting for the detected pleiotropy if any. However, it may entail sacrificing statistical power to some extent (38). On the other hand, the MR-PRESSO method can detect potential outliers and generate relatively unbiased causal estimates after removal of outliers (39). Furthermore, the embedded distortion test can be employed to evaluate the disparities between the obtained estimates before and after outlier removal (39). We also undertook the leave-one-out analysis and utilized the scatter plot to depict the associations of genetically predicted IL-1Ra concentrations with CHD/MI to examine whether a single SNP drove the association. Multivariable MR (MVMR) analysis was performed to explore the potential mechanisms and to assess whether the associations of IL-1Ra concentrations with CHD/MI changed after the adjustment for cardiometabolic risk factors (40). The two-step network MR mediation analysis was further conducted to investigate the extent to which any effect of IL-1Ra concentrations on CHD/MI might be mediated through apolipoprotein B (41). And we estimated the proportion mediated by multiplying the effect size of IL-1Ra concentrations on apolipoprotein B by that of apolipoprotein B on CHD/MI. Besides, we further investigated the causal effect of CHD and MI on IL-1Ra concentrations, considering the possibility of reverse causality. When a SNP was unavailable, we employed the SNiPa (http://snipa.helmholtz‐muenchen.de/snipa3/) to search for a proxy SNP in linkage disequilibrium (*r* ^2^>0.8) with the unavailable SNP based on the European population genotype data. The statistical significance threshold for the association of IL-1Ra concentrations with cardiometabolic risk factors was set at a two-sided *p* of <0.005 (=0.05/10 tests). Besides, the threshold for the effect of lifestyle on IL-1Ra concentrations was set at a two-sided *p* of <0.004 (=0.05/13 tests). The statistical analyses were conducted with R software (version 4.2.0) using ‘TwoSampleMR’, ‘MendelianRandomization’, and ‘MR-PRESSO’ packages (39, 42, 43).

## Results

**Figure 2:**
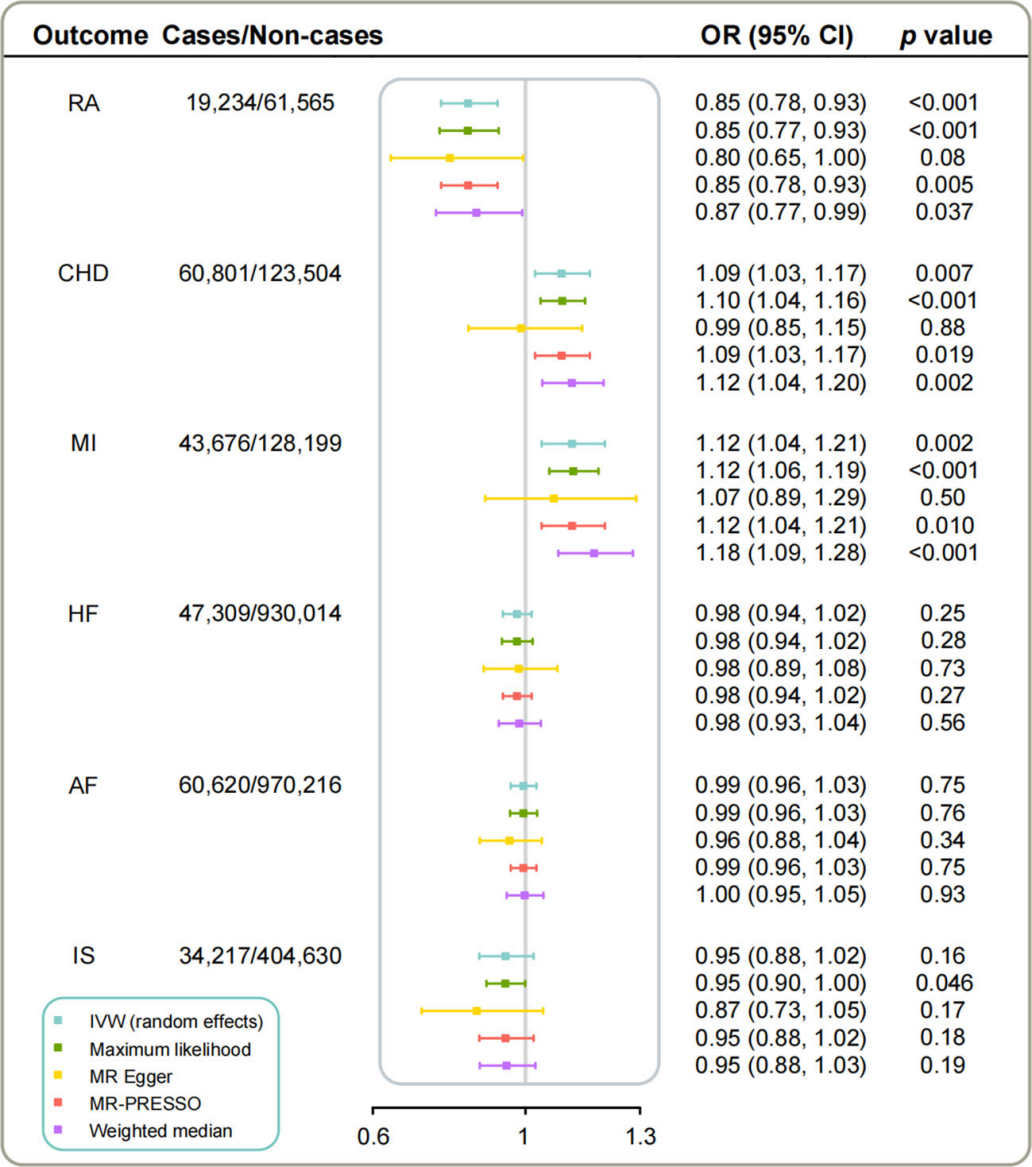
The causal associations of genetically predicted IL-1Ra level with RA and CVDs in different statistical models. Odds ratios are scaled per 1 standard deviation increase in the genetically predicted serum IL-1Ra level. IL-1Ra, interleukin 1 receptor antagonist; CHD, coronary heart disease; MI, myocardial infarction; HF, heart failure; AF, Atrial fibrillation; IS, ischemic stroke; RA, rheumatoid arthritis; OR, odds ratio; CI, confidence interval; IVW, inverse variance weighted.

The IVW analyses yielded compelling results demonstrating significant associations of genetic predicted higher concentrations of IL-1Ra with decreased risk of RA as well as decreased levels of CRP (**Figure 2**, **Table 2**). However, the corresponding risk increased by 9% and 12% per 1-SD increase in serum IL-1Ra for CHD (95%CI: 1.03-1.17; *p*=6.9×10^-3^) and MI (95%CI: 1.04-1.21; *p*=2.2×10^-3^), respectively (**Figure 2**). The sensitivity analyses indicated that MR associations of IL-1Ra with CHD/MI risk kept consistent in the majority of statistical methods, supporting the robustness of our findings, albeit with wider 95% CIs in the MR-Egger regression (**Figure 2**, **Supplementary Table 3**). No consistent evidence was found for the causal effect of genetic predicted serum IL-1Ra level on HF, AF or IS (**Figure 2**). Neither MR-PRESSO nor MR-Egger intercept test detected potential directional pleiotropy. The results of the leave-one-out analysis suggested that the causal association between IL-1Ra concentration and increased risk of CHD/MI was not drastically driven by any individual variant (**Supplementary Figures 1–6**).

**Table 2:** Associations of genetically determined IL-1Ra level with CRP and cardiometabolic risk factors.

	Q	*P*_Q_	IVW					Weighted Median					MR-Egger							
			OR/beta		95% CI		*P*	OR/beta		95% CI		*P*	OR/beta		95% CI		*P*		*P*_pleiotropy_	
CRP	18	0.01	-0.18		-0.22, -0.15		2.5E-24	-0.19		-0.22, -0.15		2.5E-25	-0.22		-0.30, -0.13		2.3E-03		0.39	
ApoA1	19	0.07	0.03		0.02, 0.05		8.7E-07	0.04		0.02, 0.05		8.1E-08	0.04		0.01, 0.08		0.03		0.54	
ApoB	8	0.69	0.05		0.04, 0.06		1.9E-23	0.05		0.03, 0.06		5.6E-11	0.03		0.00, 0.06		0.05		0.16	
HDL	13	0.32	0.02		0.01, 0.03		1.7E-03	0.02		0.01, 0.03		3.1E-03	0.03		0.00, 0.05		0.09		0.50	
LDL	11	0.44	0.05		0.04, 0.06		2.1E-17	0.05		0.03, 0.06		1.5E-11	0.03		0.00, 0.05		0.07		0.13	
TG	13	0.27	0.05		0.03, 0.06		4.8E-15	0.04		0.03, 0.06		9.1E-09	0.03		0.00, 0.06		0.05		0.30	
FG	11	0.54	0.02		0.01, 0.03		3.5E-04	0.01		0.00, 0.02		0.05	0.00		-0.03, 0.02		0.76		0.08	
FI	6	0.88	0.01		0.00, 0.02		0.07	0.01		-0.01, 0.02		0.42	0.00		-0.02, 0.03		0.90		0.64	
HbA1C	8	0.79	0.00		-0.01, 0.01		0.87	0.00		-0.01, 0.01		0.92	0.00		-0.02, 0.01		0.67		0.69	
SBP	27	0.01	-0.19		-0.45, 0.06		0.13	-0.18		-0.40, 0.04		0.10	-0.40		-1.02, 0.22		0.24		0.50	
DBP	34	0.00	-0.03		-0.19, 0.13		0.72	0.02		-0.11, 0.16		0.75	-0.25		-0.63, 0.14		0.24		0.25	

CRP, C-reactive protein; ApoA1, Apolipoprotein A-I; ApoB, Apolipoprotein B; HDL, high density lipoprotein; LDL, low density lipoprotein; TG, total triglyceride; FG, fasting glucose; FI, fasting insulin; SBP, systolic blood pressure; DBP, diastolic blood pressure; OR, odds ratio; CI, confidence interval. *P*_Q_, the *p* for Cochrane’s Q; *P*_pleiotropy_, the *p* of MR-Egger intercept test.

**Figure 3:**
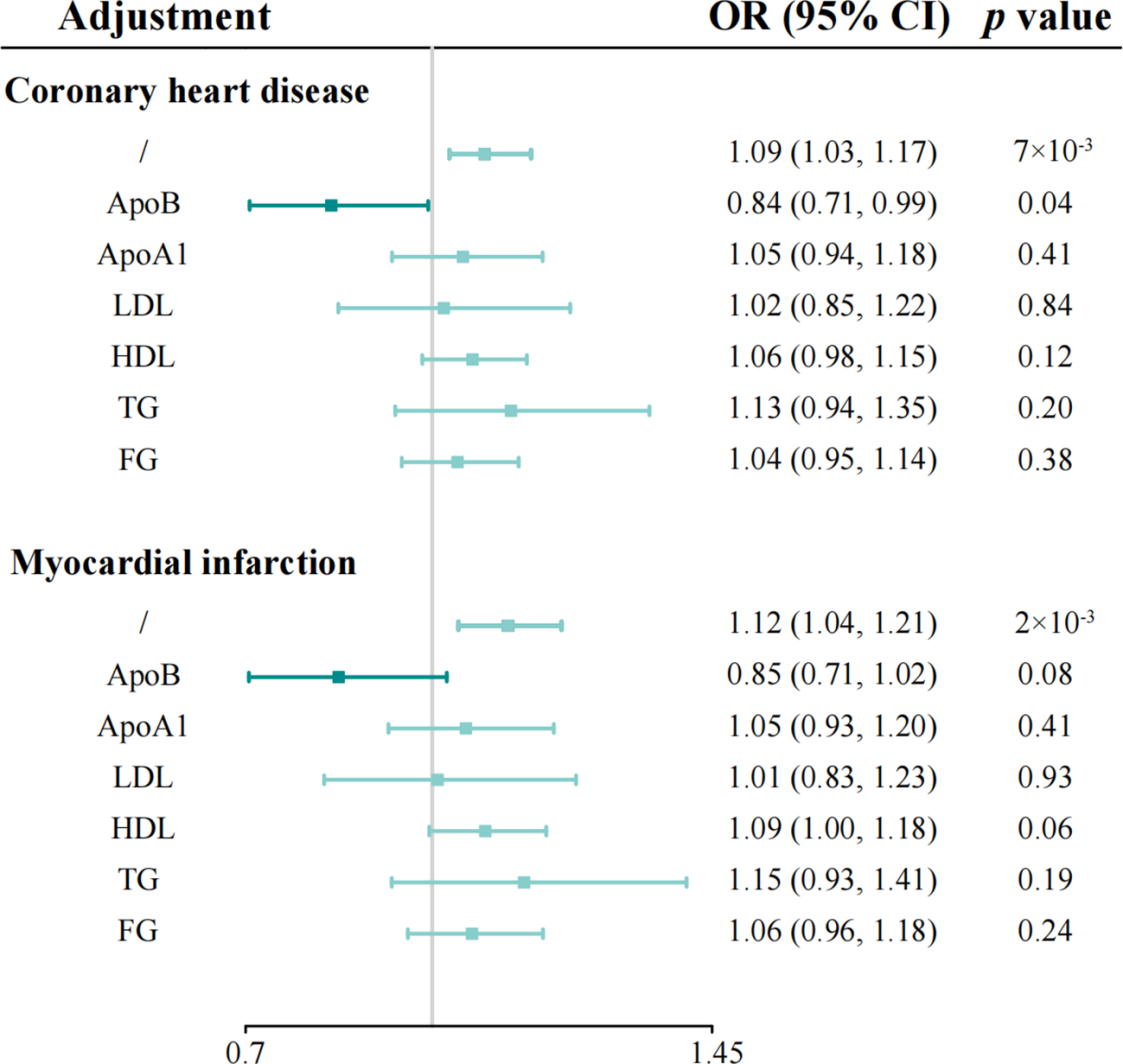
Multivariable Mendelian randomization analyses of genetically predicted IL-1Ra level with coronary heart disease and myocardial infarction on adjusting for cardiometabolic risk factors. Odds ratios are scaled per 1 standard deviation increase in the genetically predicted serum IL-1Ra level. IL-1Ra, interleukin 1 receptor antagonist; OR, odds ratio; CI, confidence interval.

**Figure 4:**
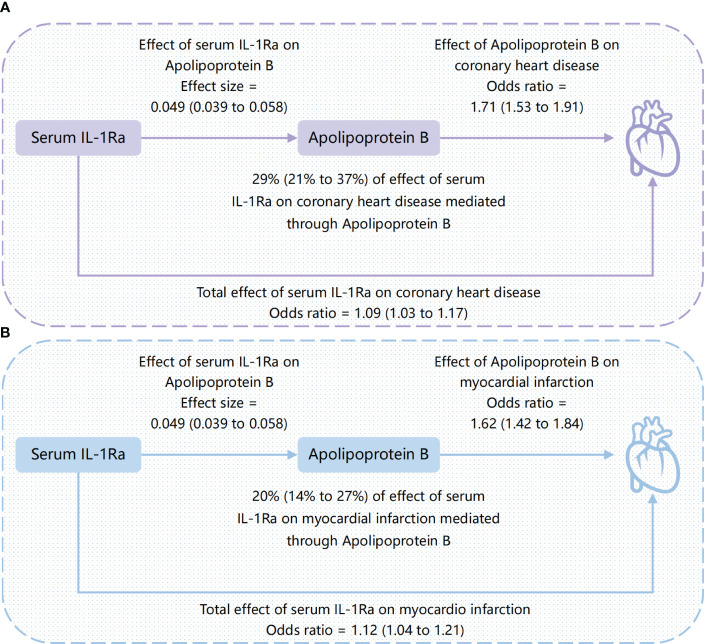
Causal directed acyclic graph showing the total effect of serum IL-1Ra on coronary heart disease and myocardial infarction, and the effect mediated by apolipoprotein B. **(A)** the mediating role of apolipoprotein B between serum IL-1Ra and coronary heart disease; **(B)** the mediating role of apolipoprotein B between serum IL-1Ra and myocardial infarction. The presented causal effect estimates with the corresponding 95% CI (shown in parenthesis) are scaled per one standard deviation increase in serum IL-1Ra. IL-1Ra, interleukin 1 receptor antagonist; CI, confidence interval.

To better understand the potential mechanisms underlying the observed causal associations, we conducted analyses examining the relationship between IL-1Ra and 10 different cardiometabolic risk factors (**Table 2**). The genetic predisposition towards a 1 SD increase in IL-1Ra concentration was found to be significantly associated with a roughly 0.05 SD increase in apolipoprotein B (effect estimate, 0.049; *p*=1.9×10^-23^), LDL (effect estimate, 0.048; *p*=2.1×10^-7^) and TG concentration (effect estimate, 0.047; *p*=4.8×10^-15^). Additionally, apolipoprotein A-I (effect estimate, 0.034; *p*=8.7×10^-7^), HDL (effect estimate, 0.017; *p*=1.7×10^-3^) and FG (effect estimate, 0.017; *p*=3.5×10^-4^) were also closely linked with IL-1Ra concentration. However, no evidence was found to support the association of IL-1Ra concentration with blood pressure. We then reassessed the association between IL-1Ra and CAD/MI with adjustment for genetically predicted cardiometabolic traits (**Figure 3**). In the MVMR with adjustment for apolipoprotein A-I, LDL, HDL and FG, the effect estimates were slightly attenuated, compared to the primary results without adjustments. Interestingly, we found that the association pattern between IL-1Ra concentration and the risk of CHD/MI drastically changed on accounting for apolipoprotein B concentrations. The direction of causal effect was reversed. After controlling for apolipoprotein B levels, suggestive evidence was found for the association between genetically predicted higher IL-1Ra concentrations and reduced risk of CHD (OR, 0.84; 95%CI: 0.71-0.99; *p*=0.04). While the association between IL-1Ra concentrations and MI was not significant (OR, 0.85; 95%CI: 0.71-1.02; *p*=0.09), with the adjustment for apolipoprotein B. The two-step mediation analysis indicated that 29% (95%CI: 21% to 37%) and 20% (95%CI: 14% to 27%) of the detrimental effect of serum IL-1Ra on CHD and MI was mediated through apolipoprotein B concentrations, respectively (**Figure 4**). Reverse-direction analyses were also performed to assess the potential reverse causality, and we found no significant evidence for the reverse causal effect of CHD or MI on the serum IL-1Ra levels (**Supplementary Tables 4–7**, **Supplementary Figures 7–12**).

We further investigated the associations of obesity and 11 lifestyle factors on serum IL-1Ra concentrations (**Supplementary Figure 13**). Body mass index, waist circumference, sedentary behaviour, and insomnia were positively associated with increased serum concentrations of IL-1Ra. On the other hand, suggestive evidence was found for the inverse association between sleep duration and IL-1Ra concentrations. No significant associations were detected for other studied lifestyle factors with IL-1Ra concentrations. In the mediation analysis, we found that adjusting for IL-1Ra concentrations led to a 47.8% attenuation of the causal effect estimate of sleep duration on the risk of CHD, and a 15.6% and 13.1% attenuation for smoking index and sedentary behaviour, respectively (**Supplementary Figure 14**).

## Discussion

The current study employed a comprehensive framework of MR analyses based on large-scale human genetic data to investigate the causal associations of long-term IL-1 inhibition with the cardiometabolic profile. We found a significant positive association between serum IL-1Ra concentration and the risk of developing CHD and MI, as well as circulating levels of lipoprotein lipids, apolipoproteins and FG. Notably, the observed association between IL-1Ra and CHD/MI was primarily driven by apolipoprotein B. No evidence was found for the link of serum IL-1Ra with HF, AF or IS. Obesity and several unhealthy lifestyle factors, like sedentary behaviour and insomnia were associated with increased concentrations of IL-1Ra. Mediation analysis suggested that IL-1Ra might mediate half of effects of a short sleep duration on CHD risk. These findings on the other side imply that in addition to drug treatment, appropriate lifestyle intervention helps to lower IL-1Ra concentration and consequently mitigate the risk of CHD.

Our findings were in line with a meta-analysis of six population-based cohort studies, involving over 20,000 participants, which revealed that the risk of CVD increased by about 11% (95% CI, 1.06-1.17), for every 1 SD increase in serum IL-1RA (5). Furthermore, the current study revealed the long‐term and stable effect of lifetime exposure to elevated serum IL-1RA level. This observation was in agreement with our previous research, which demonstrated a causal association between IL-1RA concentration and CHD risk (12). Additionally, an early MR study suggested that long-term dual inhibition of IL-1α/β elevated LDL-cholesterol concentration, but not apolipoprotein or glycaemic traits (13). Nevertheless, our study provided consistent evidence for the causal effects of serum IL-1RA levels on apolipoprotein and FG. More importantly, we discovered that apolipoprotein B played a key role in mediating and potentially reversing the relationship between the IL-1RA and the risk of developing CHD, which may pave the way for new approaches to preventing and treating this condition.

The role of IL-1RA in incidence and development of CHD is not fully understood and remains controversial. On one hand, IL-1RA is known to bind to the IL-1 receptor, thereby preventing the binding of both IL-1α and IL-1β, and it has been proposed that inhibiting the IL-1 pathway may prevent the development of various CVDs, including atherosclerosis, MI, and heart failure (7, 44). On the other hand, it has been indicated that IL-1 can directly affect lipid metabolism by suppressing the activity of lipoprotein lipase (45). Observational studies revealed a positive and significant correlation between IL-1RA and several molecules related to lipid metabolism, including cholesterol, triglycerides, and apolipoproteins B, all of which are known risk factors for CHD (5, 45). Apolipoprotein B has been postulated as a key factor in the development of atherosclerosis, likely through the “response to retention” hypothesis (46). Lipoprotein particles, particularly those containing apolipoprotein B, become trapped in the innermost layer of arterial walls. The size and composition of apolipoprotein B particles, as well as the number of particles present in the bloodstream, would influence the tendency to be trapped in the arterial wall and contribute to the development of atherosclerosis (46). Genetic evidence also suggested that apolipoprotein B was the predominant lipids trait and may play a dominant role in the development of CHD (22). The effect estimates of other blood lipids were substantially attenuated, when adjusting for apolipoprotein B (22).

Besides, the IL-1 receptor is widely distributed across human cells and plays crucial roles in numerous physiological processes, including host defence, wound healing, and autoimmunity, to name a few (4). Both IL-1α and IL-1β have been shown to have potentially beneficial cardiovascular effects, which might be hindered by the presence of IL-1Ra. Interestingly, an investigation conducted on an IL-1 receptor-deficient mouse indicated the cardioprotective effects of IL-1 signalling in advanced atherosclerosis (47). IL-1α, expressed by various cell types, not only mediates inflammation-related functions but also serves as an autocrine growth factor. IL-1β has also been shown to promote atheroprotective changes in advanced atherosclerotic lesions, such as facilitating outward remodelling and aiding in the formation and maintenance of the fibrous cap rich in smooth muscle cells and collagen (48).

The link between lifestyle and the IL-1 pathway in CVDs is compelling. Emerging evidence suggests that an unhealthy lifestyle, such as a diet high in fat and calories, physical inactivity, sleep deficit and chronic stress, can markedly upregulate IL-1 production, triggering a cascade of pro-inflammatory responses (49, 50). Sleep deprivation has been found to upregulate the expression and production of IL-1β in circulation and peripheral tissues across a variety of species, including humans, mice, rats, rabbits, cats, and monkeys, which underscores the critical role of sleep in regulating immune responses and inflammatory signaling pathways (51). Growing evidence suggests that regular physical activity exerts an overall anti-inflammatory effect by stimulating the release of muscle-derived myokines that enhance the production of IL-1Ra and IL-10, reducing dysfunction in adipose tissue and enhancing oxygen delivery (50). Cigarette smoking has also been linked to a diverse array of changes in immune and inflammatory markers, including IL-1RA, IL-1β and CRP, particularly among older individuals with a history of prolonged smoking (52).

The present study employed a comprehensive MR approach to investigate the causal relationship and underlying mechanism between IL-Ra and CHD. The study design enabled unbiased estimation of causal effects from observational data and sensitivity analyses. Moreover, the potential interaction between various lifestyle factor and serum IL-Ra was further investigated. However, certain limitations should be acknowledged. First, the possibility of horizontal pleiotropy cannot be entirely ruled out, although this was not detected in MR-Egger intercept test and MR-PRESSO analysis. Second, the current study was restricted to individuals of European ancestry. Thus, the generalizability of the results to other populations may be limited. Besides, the research population of current study was not exclusively of European descent. Third, the magnitude and duration of inhibition may differ between genetic and pharmacological IL-1 inhibition, and the findings need to be further investigated. Furthermore, the current study was limited to the use of summary-level data, and therefore, an investigation into the potential differences in the association patterns between sexes was not possible due to the lack of available sex-specific data.

## Conclusion

In summary, this study yields compelling evidence for the causal link between genetically predicted serum IL-1Ra and elevated risk of CHD/MI, with apolipoprotein B as the key driver, and a potential target for reversal. The potential cardiovascular benefits of a combined therapy involving IL-1 inhibition and lipid-modifying treatment aimed at apolipoprotein B merit further exploration, especially in individuals received IL-1Ra treatment, such as patients with RA. IL-1 pathway appeared to mediate the associations between unhealthy lifestyle factors and CHD risk, providing potential therapeutic targets for individuals with an unhealthy lifestyle to reduce CHD risk.

## Data availability statement

The original contributions presented in the study are included in the article/**Supplementary Files**, further inquiries can be directed to the corresponding author/s.

## Ethics statement

Ethical approval was not required for the studies involving humans because all studies included in the GWAS had obtained ethical approval from the appropriate committees and written informed consent had been obtained from all participants. The data used in the current study were publicly available. The studies were conducted in accordance with the local legislation and institutional requirements. The participants provided their written informed consent to participate in this study.

## Author contributions

FY: Conceptualization, Investigation, Methodology, Software, Writing – original draft. NH: Conceptualization, Investigation, Software, Supervision, Writing – original draft. JS: Resources, Validation, Visualization, Writing – original draft. PS: Data curation, Formal Analysis, Validation, Writing – review & editing. LZ: Data curation, Resources, Validation, Writing – review & editing. HC: Funding acquisition, Resources, Supervision, Writing – review & editing. SY: Conceptualization, Investigation, Methodology, Software, Supervision, Writing – original draft.
