# Supplementary material for: Apolipoprotein B and interleukin 1 receptor antagonist: reversing the risk of coronary heart disease
Source: Front Endocrinol (Lausanne). 2023 Oct 24;14:1278273. doi: 10.3389/fendo.2023.1278273 (PMC10628700; doi:10.3389/fendo.2023.1278273)

Supplementary materials

Supplemental Table 1. Single nucleotide polymorphisms used as genetic instruments for serum interleukin 1 receptor antagonist level.

SNP	Chr	Pos	EA	OA	EAF	Beta	Se	*P*-value	r^2	*F*- statistics
rs1143636	2	113589374	A	G	0.9753	0.242	0.038	1.8E-10	0.3%	88
rs4591347	2	113658705	T	C	0.1351	-0.101	0.015	1.7E-11	0.2%	73
rs7584409	2	113742734	A	G	0.7119	-0.086	0.012	9.5E-14	0.3%	94
rs115652897	2	113790332	T	C	0.0187	-0.251	0.043	3.7E-09	0.2%	72
rs114189538	2	113821082	A	G	0.066	-0.167	0.022	6.2E-14	0.3%	106
rs6743376	2	113832333	A	C	0.6472	-0.182	0.011	3.5E-65	1.5%	476
rs139076984	2	113839374	T	G	0.9786	0.230	0.040	9.8E-09	0.2%	68
rs6734238	2	113841030	A	G	0.6122	0.204	0.010	2.5E-85	2.0%	623
rs74667587	2	113895079	A	G	0.1806	-0.149	0.014	2.7E-26	0.7%	206
rs72829856	2	113913287	A	G	0.0953	0.213	0.020	3.0E-27	0.8%	243
rs1491585	2	114003621	A	G	0.5254	-0.075	0.011	2.0E-12	0.3%	87
rs1191686	2	114094703	T	C	0.1868	-0.072	0.013	4.3E-08	0.2%	49
rs1867868	2	114162983	T	C	0.4907	0.063	0.011	2.2E-09	0.2%	62

Note: SNP, single nucleotide polymorphism; Chr, chromosome; Pos, position; EA, effect allele; OA, other allele; EAF, effect allele frequence; Se, standard error.

Supplemental Table 2. Detailed information on data sources for lifestyle factors.

Trait	Unit	Participants	Ancestry	Adjustments	PubMed ID
Body mass index	SD (~ 4.8 kg/m2)	806,834 individuals	European	Age, sex, and genetic 1–5 principal components	30239722
Waist circumference	SD	224 459 individuals	European	Age and study-specific covariates	25673412
Smoking initiation	SD in prevalence of smoking initiation	1,232,091 individuals	European	Age, sex, and the first ten genetic principal components	30643251
Lifetime smoking index	SD change of lifetime smoking index	462,690 individuals	European	Genotyping chip and sex	31689377
Alcohol drinking	SD increase of log-transformed alcoholic drinks/week	941,280 individuals	European	Age, sex, and the first ten genetic principal components	30643251
Alcohol dependence	SD in the prevalence of alcohol dependence	46,568 individuals	European	Sex and 1-5 principal components	30482948
Coffee consumption	50% change	375 ,833 individuals	European	Age, sex, body mass index,	31046077

				total energy, proportion of typical food intake, and 20 genetic principal components	
Caffeine consumption	80 mg increase (equivalent to dose from 1 cup of coffee)	9,876 individuals	European	Age, sex, study-site, fasting status, smoking status, and genetic principal components	27702941
moderate-to-vigorous PA	MET-minutes/week	377,234 individuals	European	Age, sex, genotyping chip, first ten genomic principal components, and center	29899525
Vigorous physical activity	≥ 3 versus 0 day/week	98,060 cases and 162,995 controls	European	Age, sex, genotyping chip, first ten genomic principal components, and center	29899525
Sedentary behavior	SD (~1.5 hours) change of leisure television watching time	408,815 individuals	European	Age-squared, age, sex, age-sex interaction, and the first 30 principal components	32317632
Sleep duration	60 minutes	446,118 individuals	European	Age, sex, and 10 principal	30846698

				components	
Insomnia	SD in the prevalence of insomnia	397,959 cases and 933,057 controls	European	age, sex, genotype array, and 10 genetic principal components in the UK Biobank; age, sex and the top five principal components in 23andMe	30804565

Supplemental Table 3. Associations of Genetically Determined IL1Ra Level with CHD, MI and Cardiometabolic Risk Factors in MR-PRESSO Analyses.

	OR	95% CI	P	Outlier	$P_{\text{distrotion}}$
CHD	NA	NA	NA	0	NA
MI	NA	NA	NA	0	NA
ApoA1	NA	NA	NA	0	NA
ApoB	NA	NA	NA	0	NA
HDL	NA	NA	NA	0	NA
LDL	NA	NA	NA	0	NA
TG	NA	NA	NA	0	NA
FG	NA	NA	NA	0	NA
FI	NA	NA	NA	0	NA
HbA1C	NA	NA	NA	0	NA
SBP	-0.24	-0.44, -0.04	0.04	1	0.66
DBP	-0.07	-0.18, 0.05	0.29	1	0.72
CRP	NA	NA	NA	0	NA
RA	NA	NA	NA	0	NA

Note: CHD, coronary heart disease; MI, myocardial infarction; ApoA1, Apolipoprotein A-I; ApoB, Apolipoprotein B; HDL, high density lipoprotein; LDL, low density lipoprotein; TG, total triglyceride; FG, fasting glucose; FI, fasting insulin; SBP, systolic blood pressure; DBP, diastolic blood pressure; CRP, C-reactive protein; RA, rheumatoid arthritis; OR, odds ratio; CI, confidence interval. $P_{\text{distrotion}}$, the p value of MR-PRESSO distrotion test.

Supplemental Table 4. The Genetic Associations for Coronary Heart Disease and Serum Interleukin 1 Receptor Antagonist Level.

SNP	beta.exposure	se.exposure	beta.outcome	se.outcome
rs10139550	-0.055	0.010	0.003	0.008
rs10840293	0.055	0.010	0.013	0.008
rs11065979	-0.069	0.011	-0.047	0.008
rs11191416	0.079	0.014	0.006	0.013
rs11206510	0.075	0.013	-0.005	0.011
rs11556924	0.073	0.011	0.012	0.008
rs115654617	-0.138	0.016	0.012	0.013
rs11617955	0.089	0.016	-0.013	0.012
rs11838776	-0.069	0.011	-0.001	0.009
rs1199338	-0.074	0.012	0.011	0.011
rs12202017	0.067	0.010	0.000	0.011
rs1412444	-0.067	0.010	-0.015	0.008
rs16986953	-0.085	0.015	-0.015	0.017
rs17087335	-0.061	0.011	-0.002	0.010
rs17678683	-0.099	0.017	-0.041	0.012
rs180803	-0.181	0.028	-0.024	0.032
rs186696265	-0.550	0.048	0.026	0.040
rs1870634	0.076	0.010	-0.013	0.009
rs2107595	-0.073	0.011	0.007	0.011
rs2128739	-0.066	0.010	0.001	0.009
rs2487928	-0.063	0.010	0.007	0.008
rs2681472	-0.074	0.011	-0.017	0.010
rs28451064	-0.128	0.016	0.006	0.012
rs2891168	-0.193	0.009	0.004	0.008
rs3918226	-0.133	0.022	0.000	0.013
rs41290120	0.181	0.028	-0.048	0.020
rs4420638	-0.092	0.014	0.044	0.010
rs4468572	0.077	0.010	-0.006	0.008
rs4593108	0.071	0.012	0.003	0.010
rs4773141	-0.070	0.012	-0.018	0.008
rs515135	0.067	0.012	-0.011	0.012
rs55730499	-0.317	0.024	0.043	0.017
rs56062135	0.070	0.012	0.012	0.009
rs56289821	0.134	0.017	-0.002	0.014
rs56336142	0.067	0.012	-0.011	0.010
rs663129	-0.058	0.011	-0.035	0.009
rs6689306	-0.056	0.009	-0.006	0.008

rs7212798	-0.080	0.014	0.021	0.011
rs7528419	0.115	0.011	-0.006	0.010
rs7568458	-0.060	0.010	0.030	0.008
rs8042271	-0.097	0.018	0.001	0.020
rs9349379	-0.132	0.010	-0.006	0.008
rs9970807	0.126	0.017	0.014	0.015

Note: SNP, single nucleotide polymorphism; Se, standard error.

Supplemental Table 5. The Genetic Associations for Myocardial Infarction and Serum Interleukin 1 Receptor Antagonist Level.

SNP	beta.exposure	se.exposure	beta.outcome	se.outcome
rs10176176	0.065	0.010	-0.025	0.008
rs10455872	0.285	0.027	-0.043	0.017
rs10947786	-0.072	0.013	0.012	0.010
rs113113862	-0.075	0.013	-0.001	0.009
rs11556924	-0.069	0.013	-0.012	0.008
rs1332329	0.079	0.011	0.016	0.008
rs180803	-0.187	0.032	-0.024	0.032
rs186696265	0.530	0.053	-0.026	0.040
rs1870634	0.070	0.011	-0.013	0.009
rs2019090	-0.065	0.011	0.001	0.009
rs2327426	-0.063	0.011	-0.002	0.009
rs2505083	0.061	0.011	-0.005	0.008
rs2681472	0.073	0.013	0.017	0.010
rs28451064	0.122	0.018	-0.006	0.012
rs35700460	0.082	0.012	-0.001	0.009
rs41290120	-0.186	0.031	0.048	0.020
rs429358	0.096	0.017	-0.040	0.011
rs4773141	0.080	0.013	0.018	0.008
rs4977574	0.189	0.010	-0.004	0.008
rs653178	-0.077	0.012	-0.045	0.008
rs7173743	-0.064	0.010	0.006	0.008
rs72689147	-0.074	0.013	-0.003	0.010
rs72934535	0.141	0.019	-0.002	0.014
rs7528419	-0.101	0.013	0.006	0.010
rs9349379	0.131	0.011	0.006	0.008
rs9970807	-0.111	0.018	-0.014	0.015

Note: SNP, single nucleotide polymorphism; Se, standard error.

Supplemental Table 6. The reverse causal effect of Coronary Heart Disease on Serum Interleukin 1 Receptor Antagonist Level using Different Statistical Models.

Method	nsnp	Beta	se	pval
Weighted mode	43	-0.037	0.033	0.27
MR Egger	43	-0.105	0.064	0.11
IVW (multiplicative random effects)	43	-0.012	0.029	0.69
IVW (fixed effects)	43	-0.012	0.016	0.48
Simple median	43	-0.012	0.026	0.64
Weighted median	43	-0.029	0.028	0.30
MR-PRESSO (5 outlier-corrected)	38	-0.022	0.018	0.24

Note: nsnp, number of single nucleotide polymorphism; OR, odds ratio; LCI, lower confidence interval; UCI, upper confidence interval; MR, Mendelian randomization; MR-PRESSO, MR-pleiotropy residual sum and outlier.

Supplemental Table 7. The reverse causal effect of Myocardial Infarction on Serum Interleukin 1 Receptor Antagonist Level using Different Statistical Models.

Method	nsnp	Beta	se	pval
Weighted mode	26	-0.024	0.035	0.50
MR Egger	26	-0.092	0.078	0.25
IVW (multiplicative random effects)	26	-0.017	0.035	0.63
IVW (fixed effects)	26	-0.017	0.019	0.38
Simple median	26	-0.016	0.031	0.61
Weighted median	26	-0.019	0.031	0.55
MR-PRESSO (3 outlier-corrected)	23	-0.015	0.024	0.53

Note: nsnp, number of single nucleotide polymorphism; OR, odds ratio; LCI, lower confidence interval; UCI, upper confidence interval; MR, Mendelian randomization; MR-PRESSO, MR-pleiotropy residual sum and outlier.

Figure legends

Supplemental figure 1. The scatter plot for the association between serum interleukin 1 receptor antagonist level and the risk of coronary heart disease.

Supplemental figure 2. The leave-one-out analysis for the association between serum interleukin 1 receptor antagonist level and the risk of coronary heart disease.

Supplemental figure 3. The funnel plot for the association between serum interleukin 1 receptor antagonist level and the risk of coronary heart disease.

Supplemental figure 4. The scatter plot for the association between serum interleukin 1 receptor antagonist level and the risk of myocardial infarction.

Supplemental figure 5. The leave-one-out analysis for the association between serum interleukin 1 receptor antagonist level and the risk of myocardial infarction.

Supplemental figure 6. The funnel plot for the association between serum interleukin 1 receptor antagonist level and the risk of myocardial infarction.

Supplemental figure 7. The scatter plot for the reverse causal effect of coronary heart disease on serum interleukin 1 receptor antagonist level.

Supplemental figure 8. The leave-one-out analysis for the reverse causal effect of coronary heart disease on serum interleukin 1 receptor antagonist level.

Supplemental figure 9. The funnel plot for the reverse causal effect of coronary heart disease on serum interleukin 1 receptor antagonist level.

Supplemental figure 10. The scatter plot for the reverse causal effect of myocardial infarction on serum interleukin 1 receptor antagonist level.

Supplemental figure 11. The leave-one-out analysis for the reverse causal effect of myocardial infarction on serum interleukin 1 receptor antagonist level.

Supplemental figure 12. The funnel plot for the reverse causal effect of myocardial infarction on serum interleukin 1 receptor antagonist level.

Supplemental figure 13. Causal associations of genetically predicted obesity and lifestyle factors with serum IL-1Ra level. IL-1Ra, interleukin 1 receptor antagonist.

Supplemental figure 14. The causal effect of sleep duration, smoking index and sedentary behaviour on risk of coronary heart disease with or without adjustment for

serum IL-1Ra level. IL-1Ra, interleukin 1 receptor antagonist; OR, odds ratio; POC, change of the proportion after adjusting for serum IL-1Ra level.

Supplemental Figure 1.

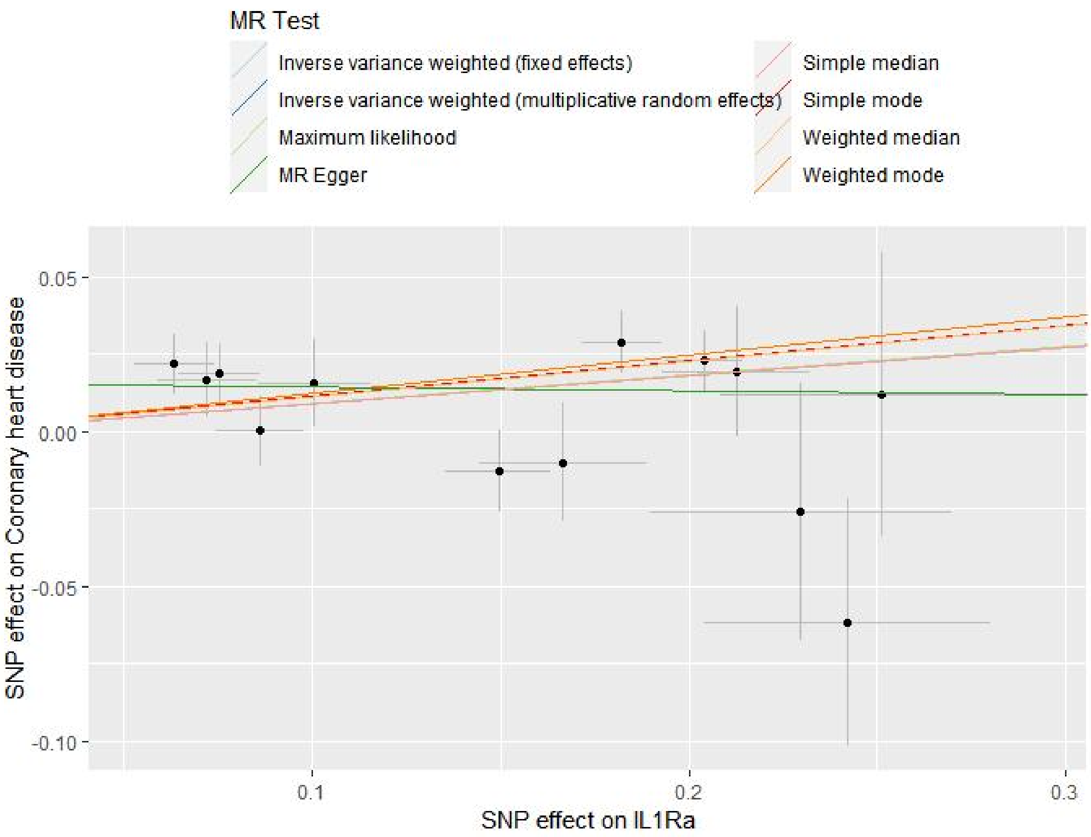

Supplemental Figure 2.

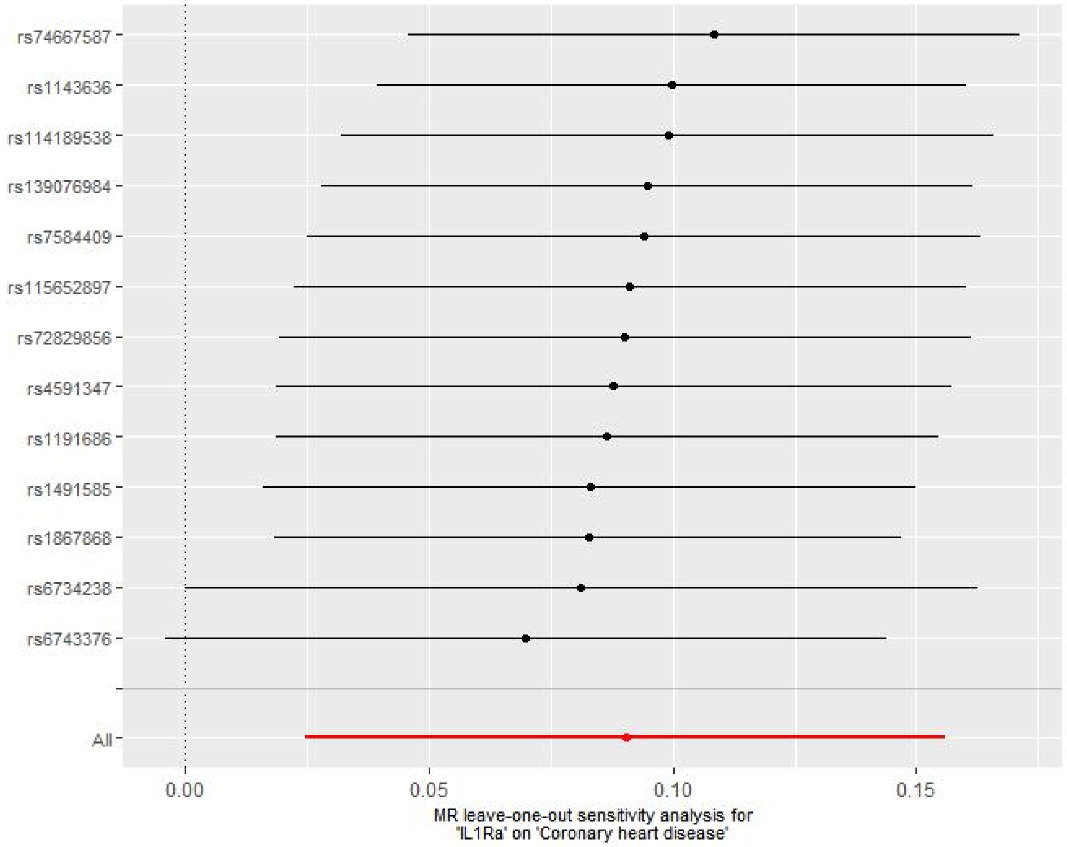

Supplemental Figure 3.

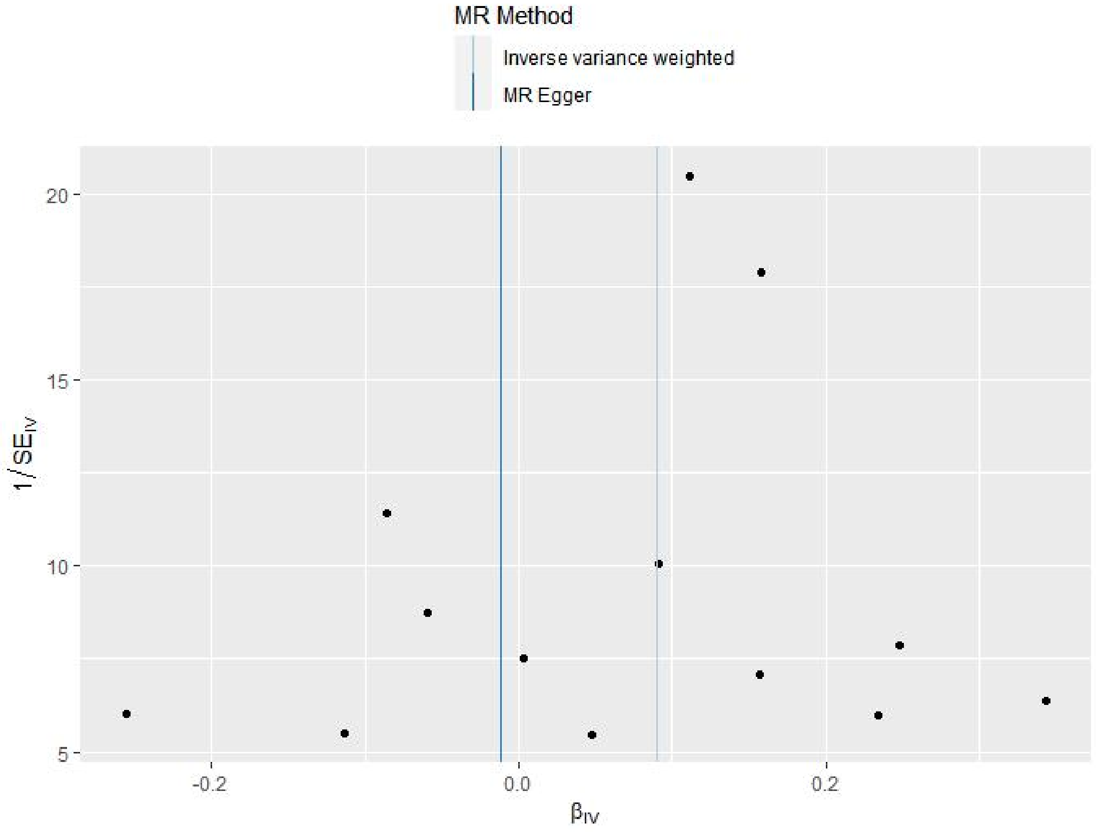

Supplemental Figure 4.

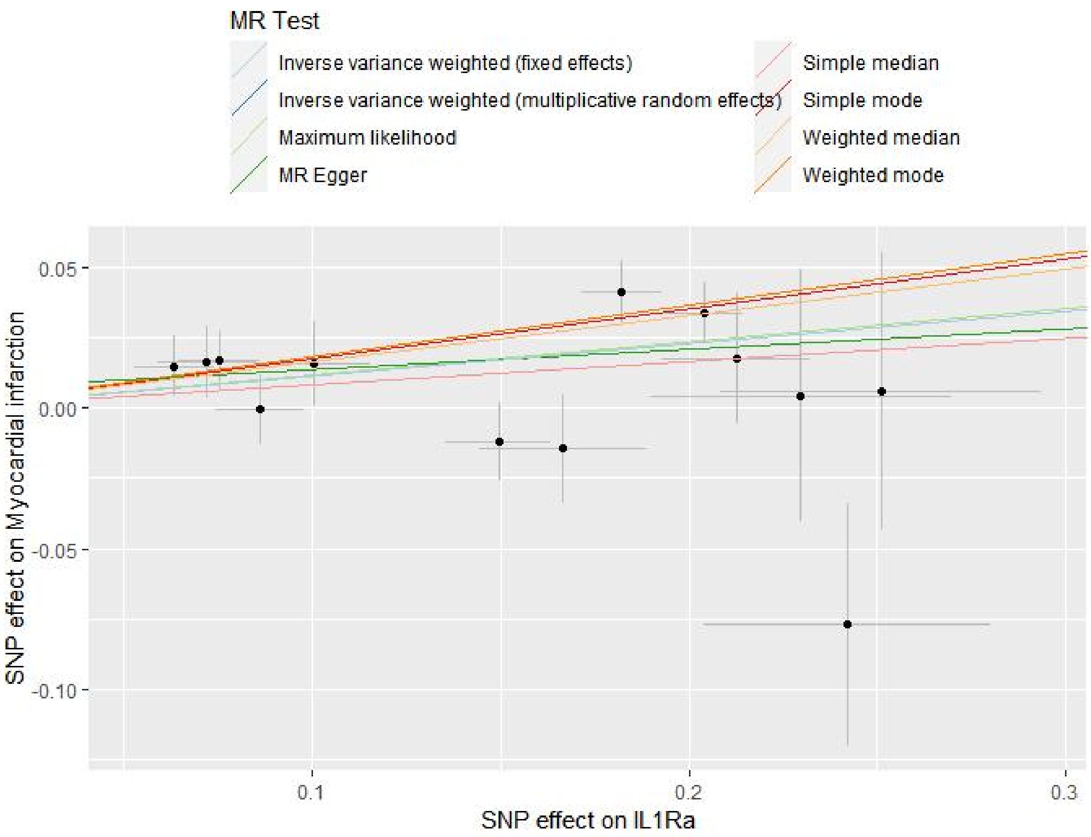

Supplemental Figure 5.

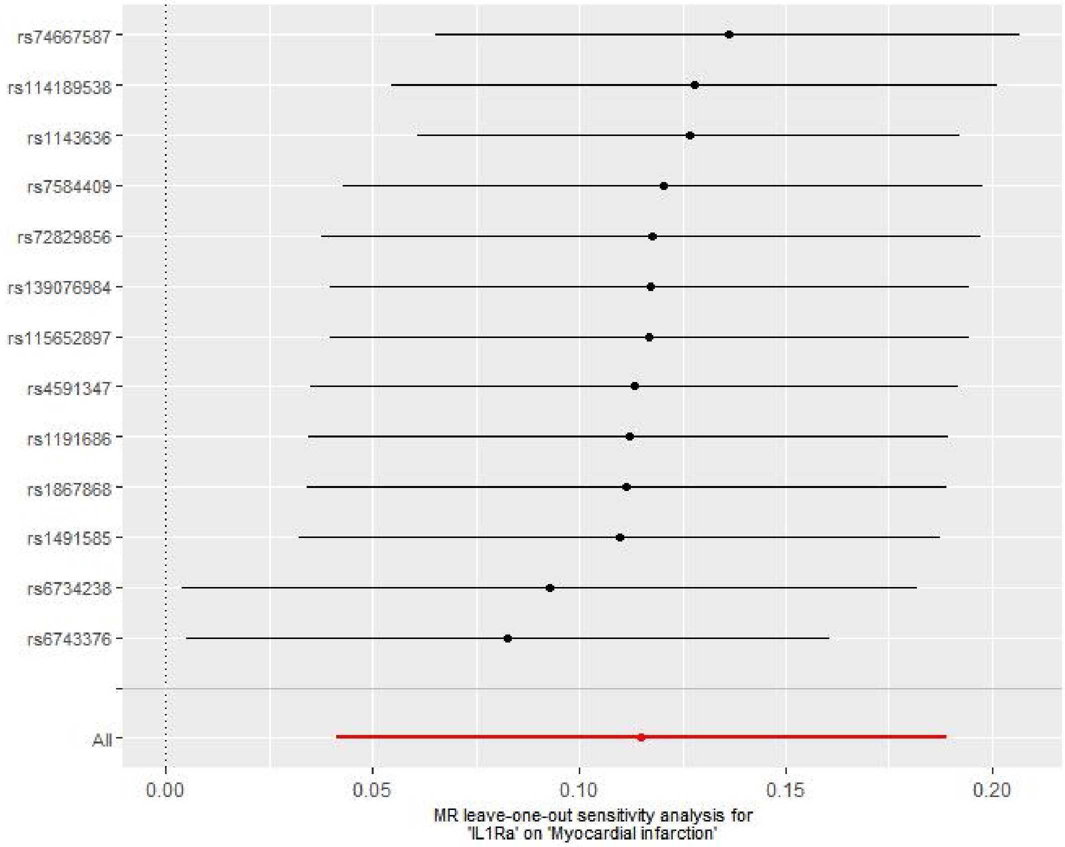

Supplemental Figure 6.

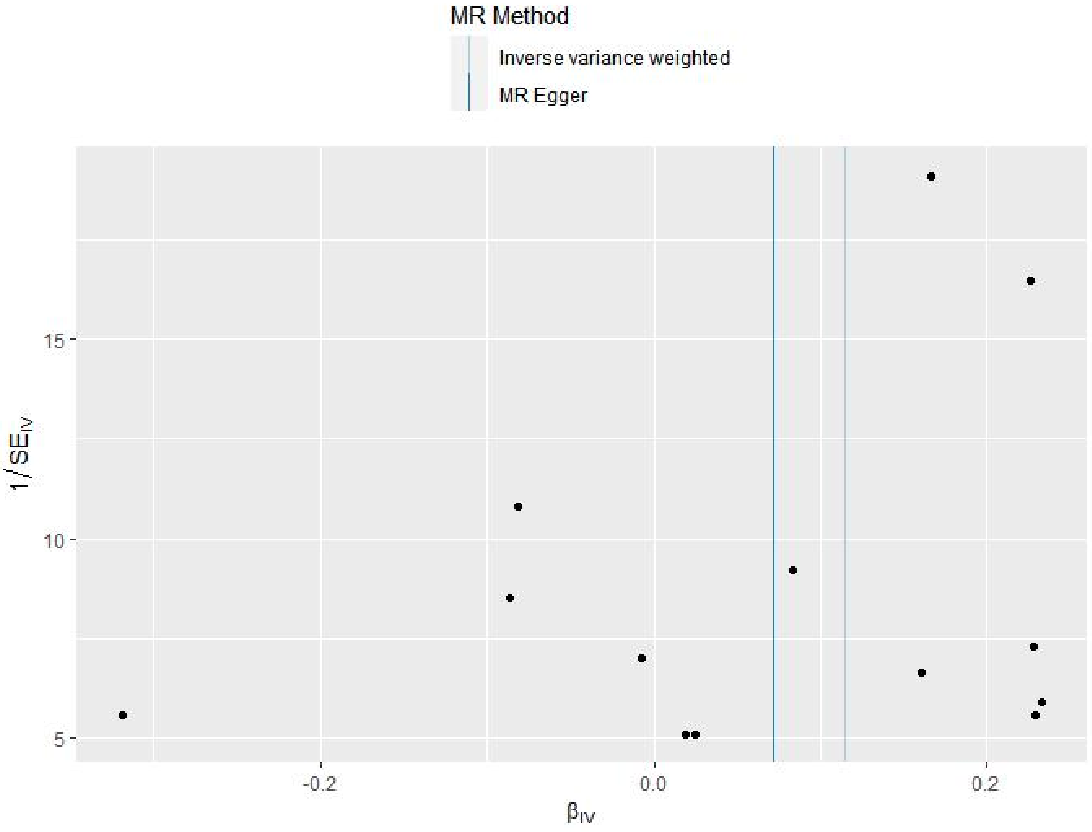

Supplemental Figure 7.

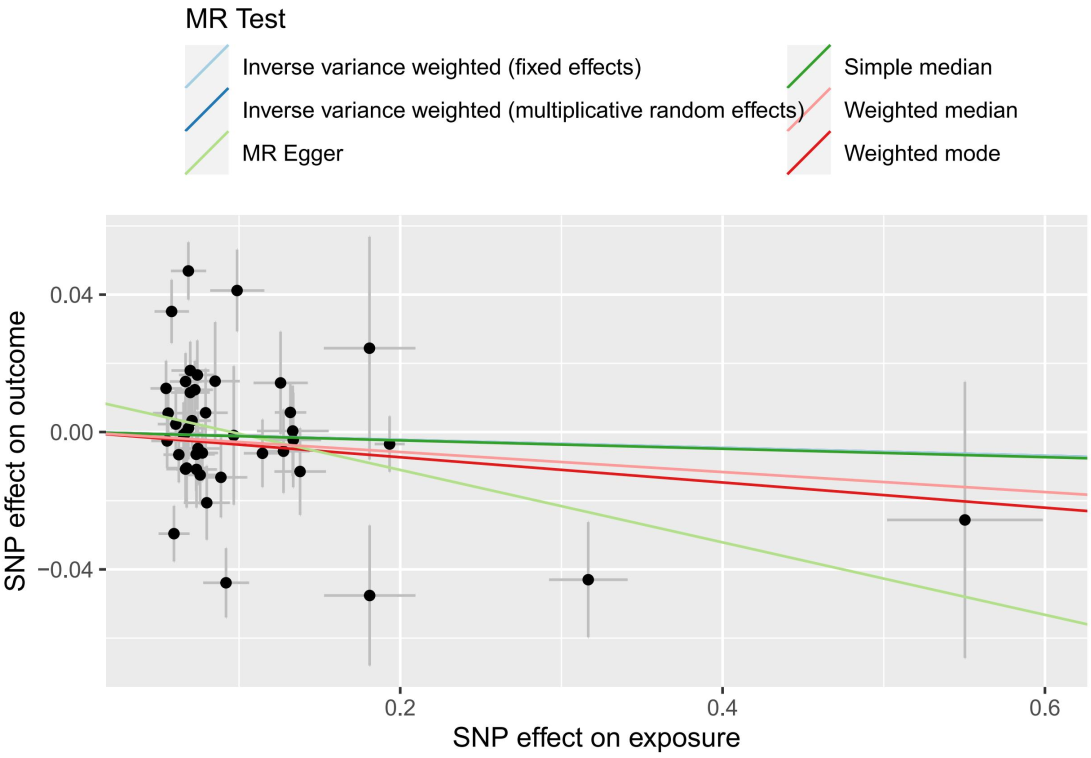

Supplemental Figure 8.

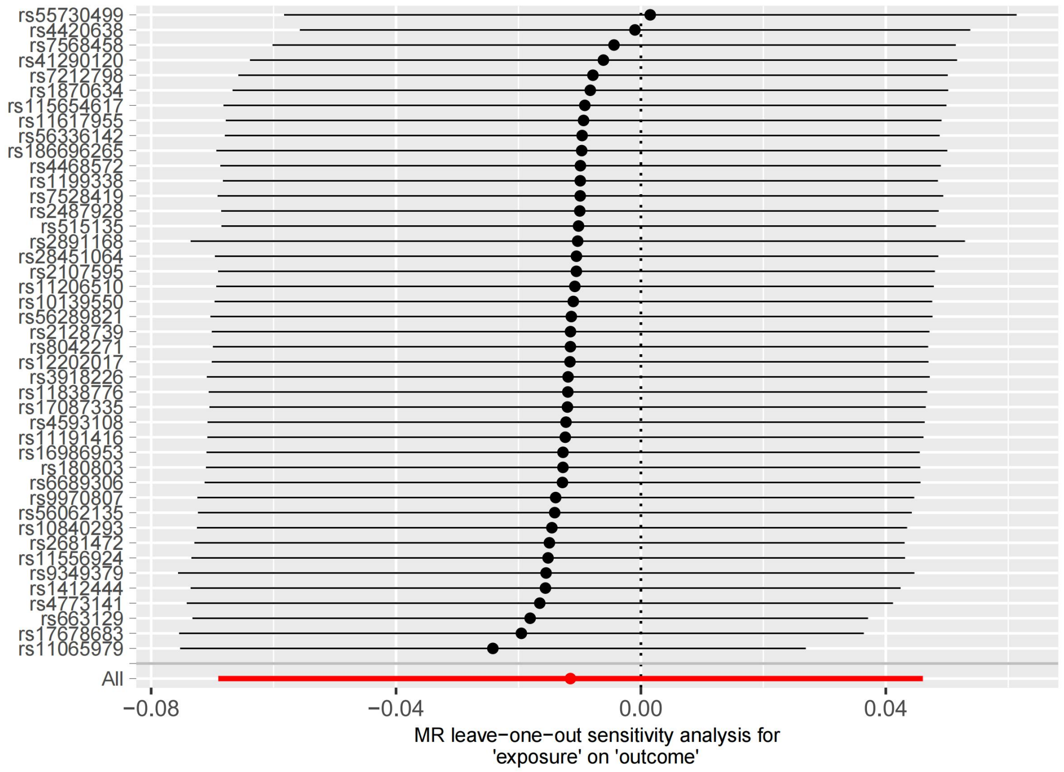

Supplemental Figure 9.

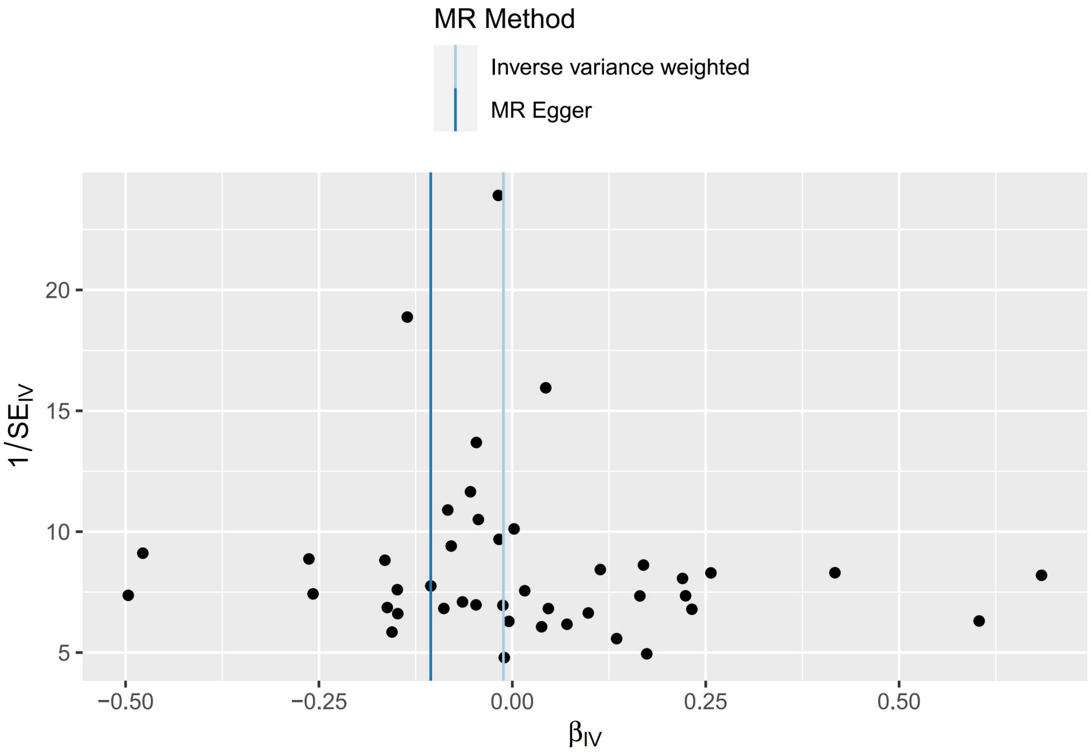

Supplemental Figure 10.

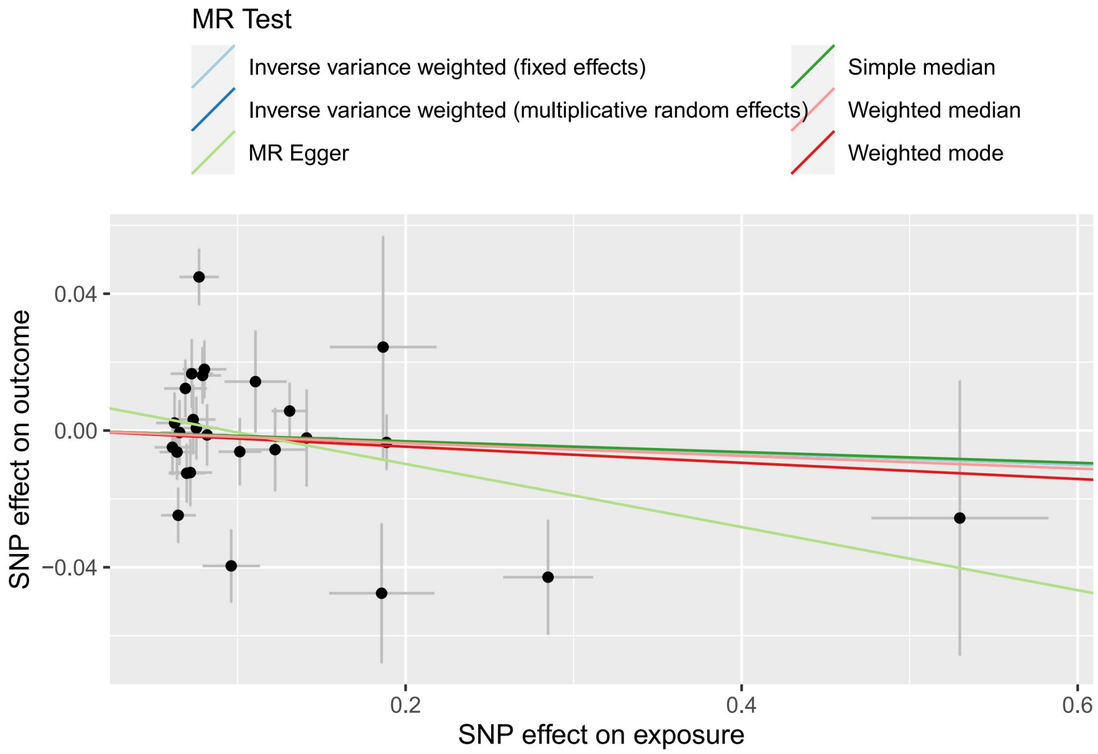

Supplemental Figure 11.

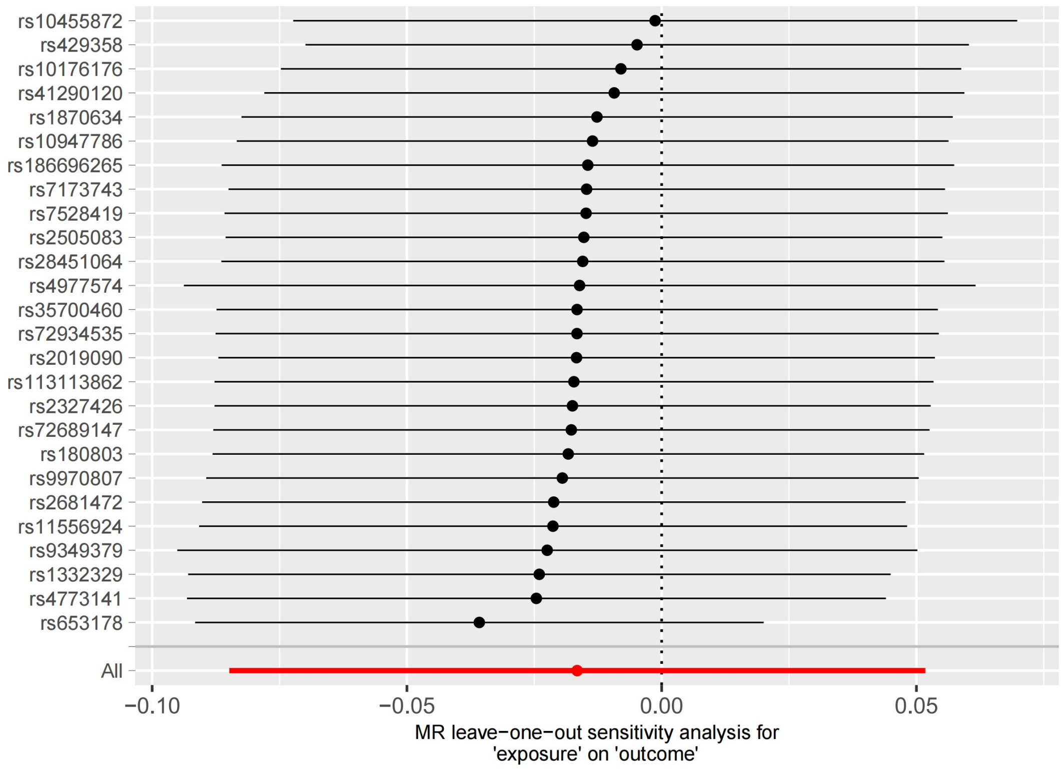

Supplemental Figure 12.

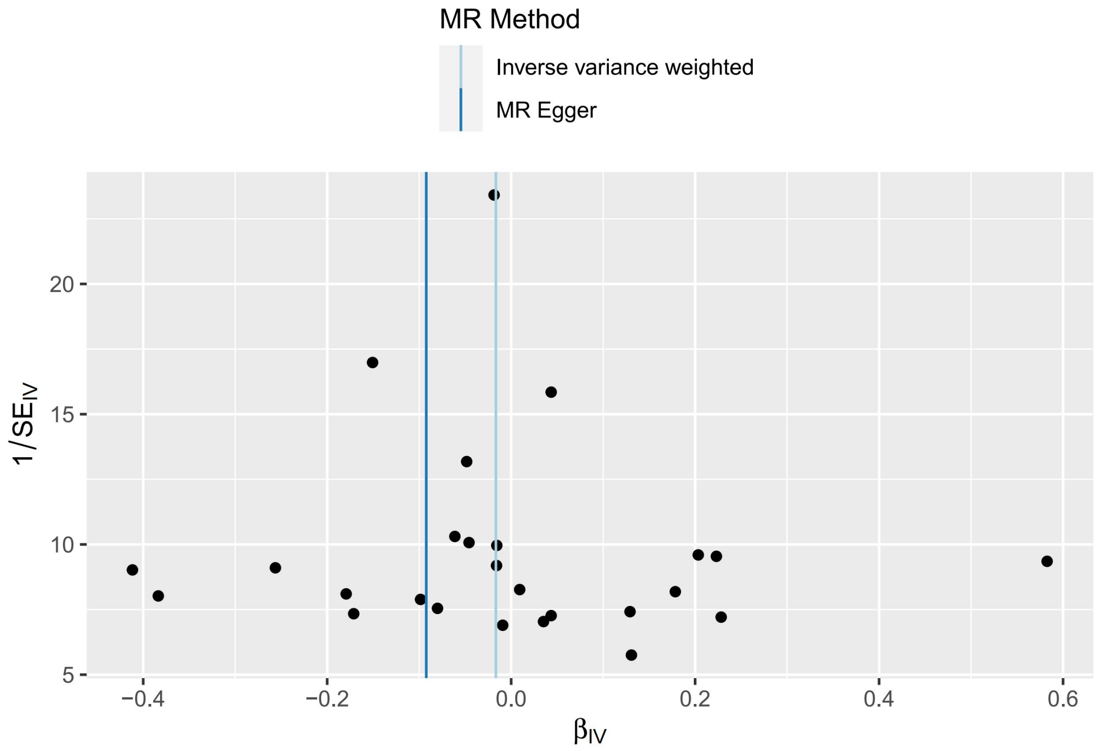

Supplemental Figure 13.

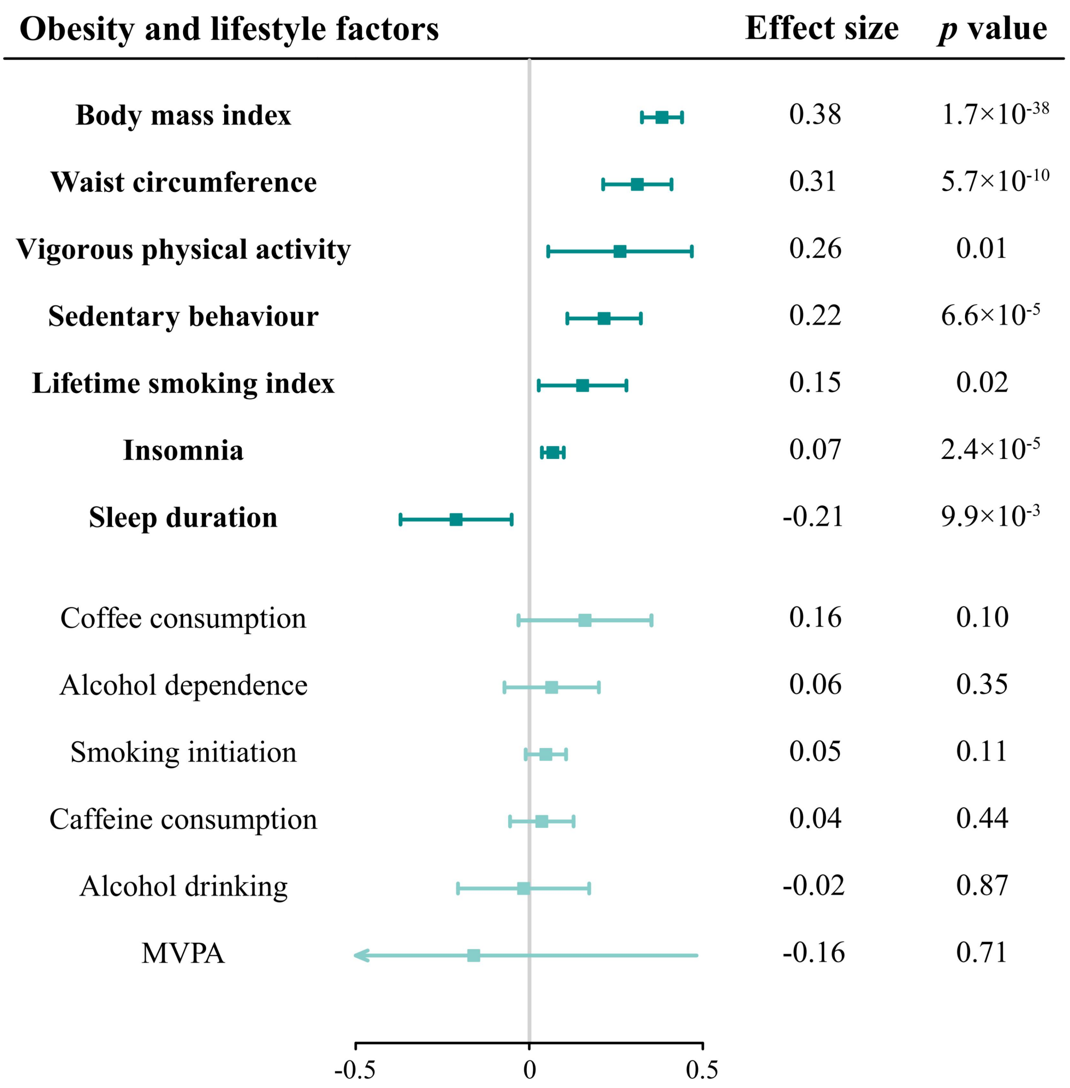

Supplemental Figure 14.

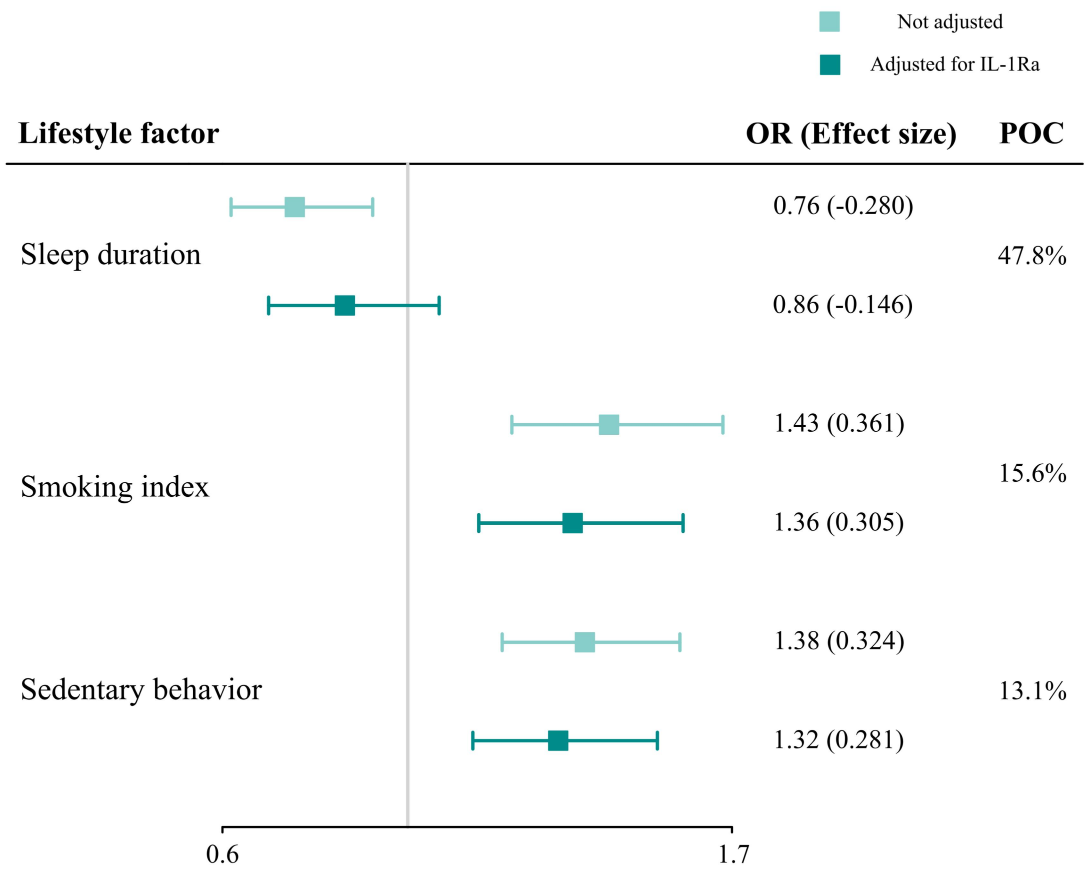

Supplement: Supplementary file 1 [file DataSheet_1.pdf]
